# Pediatric cardiorespiratory failure successfully managed with venoarterial-venous extracorporeal membrane oxygenation: a case report

**DOI:** 10.1186/s12890-016-0280-7

**Published:** 2016-08-12

**Authors:** Michihito Kyo, Shinichiro Ohshimo, Yoshiko Kida, Tatsutoshi Shimatani, Yusuke Torikoshi, Kei Suzuki, Satoshi Yamaga, Nobuyuki Hirohashi, Nobuaki Shime

**Affiliations:** Department of Emergency and Critical Care Medicine, Graduate School of Biomedical Sciences, Hiroshima University, 1-2-3 Kasumi, Minami-ku, Hiroshima 734-8551 Japan

**Keywords:** Extracorporeal membrane oxygenation, Venoarterial, Venovenous, Myocarditis

## Abstract

**Background:**

Venoarterial-venous extracorporeal membrane oxygenation (VAV ECMO) configuration is a combined procedure of extracorporeal membrane oxygenation (ECMO). The proportion of cardiac and respiratory support can be controlled by adjusting arterial and venous return. Therefore, VAV ECMO can be applicable as a bridging therapy in the transition from venoarterial (VA) to venovenous (VV) ECMO.

**Case presentation:**

We present an 11-year-old girl with chemotherapy-induced myocarditis requiring extracorporeal cardiorespiratory support. She showed progressive hypotension, tachycardia, hyperlactemia, and tachypnea under support of catecholamines. Echocardiography showed severe left ventricular hypokinesis with an ejection fraction of 30 %. She was placed on VA ECMO with a drainage catheter from the right femoral vein (19.5 Fr) and a return catheter to the right femoral artery (16.5 Fr). Extracorporeal circulation was initiated at a blood flow of 2.0 L/min (59 mL/kg/min). On day 31, although cardiac function had improved, persistent pulmonary failure made weaning from VA ECMO difficult. We planned transition from VA ECMO to VAV ECMO to ensure gradual tapering of extracorporeal cardiac support while evaluating cardiopulmonary function. An additional return cannula (13.5 Fr) was inserted from the right internal jugular vein, which was connected to the circuit branch from the original returning cannula. We then gradually shifted the blood from the femoral artery to the right internal jugular vein over 24 h. She was successfully switched from VA to VV ECMO via VAV ECMO.

**Conclusions:**

VAV ECMO might be an option in ensuring oxygenation to the coronary circulation and allowing time to adequately evaluate cardiac function during transition from VA to VV ECMO. Further investigations using larger cohorts are necessary to validate the efficacy of VAV ECMO as a bridging therapy in the transition from VA to VV ECMO.

## Background

Femoral venoarterial extracorporeal membrane oxygenation (VA ECMO) is an important tool for supporting cardiac function in patients with severe cardiac failure because of its rapid vascular access [1]. Decreased oxygenation for the coronary and right upper body circulation is one of the major disadvantages of femoral VA ECMO. This condition is particularly obvious in patients with improvement in left ventricular dysfunction and deterioration in pulmonary function. This in turn leads to deterioration in ventricular function when continuing VA ECMO. Moreover, accurate evaluation of cardiac function in patients supported by VA ECMO is sometimes difficult [2]. These factors negatively affect successful weaning from VA ECMO.

A VA-venous (VAV) ECMO configuration is a combined procedure in which ECMO enables oxygenated blood flow to the arterial and venous circulations where the proportion of flow can be easily controlled [3]. In this report, we present a pediatric patient with severe cardiorespiratory failure related to myocarditis who was managed with VAV ECMO when transitioning from VA to VV ECMO.

## Case presentation

An 11-year-old girl (140 cm in height, 34 kg in weight) was diagnosed with aplastic anemia and received hematopoietic stem cell transplantation. However, simultaneous chemotherapy using cyclosporin and cyclophosphamide induced myocarditis, resulting in cardiorespiratory failure. She presented with tachypnea (32 breaths/min), tachycardia (124 bpm), and hypotension (98/60 mmHg) on admission to the intensive care unit. Laboratory tests showed an increased N-terminal pro-B-type natriuretic peptide (NT-proBNP) level of 10,483 pg/mL (normal, <125 pg/mL) and hyperlactemia (4.5 mmol/L). A chest radiograph and computed tomography showed bilateral diffuse pulmonary infiltrate and pleural effusion.

The hemodynamic state of the patient rapidly deteriorated with progressive hypotension (87/54 mmHg), tachycardia (155 bpm), and hyperlactemia (9.1 mmol/L) under support of 4 μg/kg/min of dobutamine. Arterial blood gas analysis showed metabolic acidosis and respiratory alkalosis (pH, 7.453; partial pressure of oxygen (PaO_2_), 107 Torr; partial pressure of carbon dioxide (PaCO_2_), 27.2 Torr; HCO_3_^−^, 18.7 mmol/L; Fraction of inspired oxygen (F_I_O_2_), 0.3). Echocardiography showed severe left ventricular hypokinesis with an ejection fraction of 30 %. She was placed on VA ECMO with centrifuged pump (Capiox^TM^, Terumo, Tokyo, Japan). The drainage catheter was inserted from the right femoral vein to inferior vena cava (IVC, 19.5 Fr), and the return catheter (16.5 Fr) was inserted from the right femoral artery (FA) to the abdominal aorta. Extracorporeal circulation was initiated at a blood flow of 2.0 L/min (59 mL/kg/min) and a sweep gas flow of 1.3 L/min with an F_I_O_2_ of 1.0. The cardiorespiratory support with VA ECMO provided gradual improvement of cardiac function. On day 31, echocardiography demonstrated an improved left ventricular ejection fraction of 50 %. However, PaO_2_ of the right radial artery was 55 mmHg with a blood flow of 4.6 L/min (135 mL/kg/min), which indicated insufficient pulmonary oxygenation. Pulmonary edema and bilateral pleural effusion on chest X-ray were still evident. A trial of reducing extracorporeal blood flow up to 3.7 L/min (109 mL/kg/min) led to severe hypoxemia (blood oxygen saturation, 81 %), hypotension (50/30 mmHg), and bradycardia (60 bpm), making weaning from VA ECMO difficult. On day 32, the repeated trial of reducing extracorporeal blood flow led to severe hypotension and bradycardia again, despite the improved left ventricular ejection fraction on echocardiography and the decreased PaO_2_ of the right radial artery under extracorporeal support. We evaluated that the current cardiac function was yet insufficient without extracorporeal cardiac support, although prolonged retrograde blood flow of ECMO deteriorated an improving left ventricular function.

**Fig. 1:**
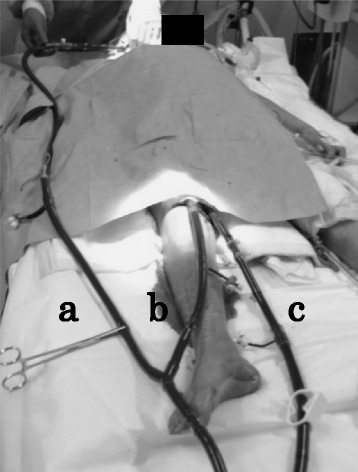
Overview of venoarterial-venous extracorporeal membrane oxygenation. **a** Photograph showing a return cannula to the right internal jugular vein; **b** a return cannula to the right femoral artery; and **c** a drainage cannula from the right femoral vein

**Fig. 2:**
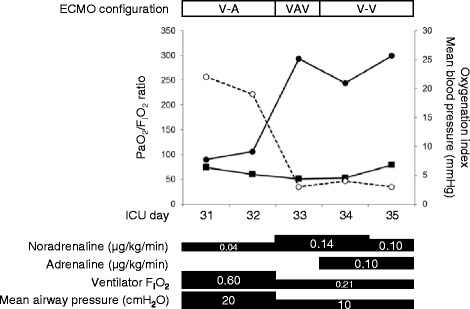
Clinical course of the patient. Graph showing the clinical course of the patient with an improvement in the PaO_2_/F_I_O_2_ ratio and oxygenation index after shifting from VA ECMO to VAV ECMO. Blood pressure was stable in shifting from VA ECMO to VAV ECMO. Closed circles indicate the PaO_2_/F_I_O_2_ ratio; open circles indicate the oxygenation index; and closed squares indicate blood pressure

On day 33, we planned transition from VA ECMO to VAV ECMO to ensure gradual tapering of extracorporeal cardiac support, because a direct transition from VA ECMO to VV ECMO had a potential risk of circulatory failure. An additional return cannula (13.5 Fr) was inserted into the right atrium via the right internal jugular vein (RIJV). This cannula was connected to the circuit branch from the original returning cannula (Fig. 1). The proportion of blood flow that returned to the right FA and RIJV was initially set as 2.0 L/min and 2.0 L/min, respectively. We then gradually shifted the blood from the FA to the RIJV over 24 h with frequent evaluation of blood pressure, right radial arterial oxygenation and left ventricular ejection fraction (Fig. 2). With a complete shift of return flow to the RIJV after 25 VAV ECMO hours, blood pressure (128/92 mmHg), heart rate (96 bpm), lactate level (3.2 mmol/L), and left ventricular ejection fraction (64 %) were stable. Arterial blood gases still showed respiratory acidosis (pH, 7.321; PaO_2_, 56.6 Torr, PaCO_2_, 60.0 Torr; HCO_3_^−^, 30.1 mmol/L).

Despite sustained management with VV ECMO, the patient died of sepsis on day 47.

## Discussion

This is the first report of pediatric cardiorespiratory failure that was managed with VAV ECMO. Transition from VA ECMO to VV ECMO was successfully performed by transposing VAV ECMO. The findings of our case indicate the clinical utility of VAV ECMO in combined cardiorespiratory failure.

Weaning from VA ECMO in patients with cardiopulmonary failure is occasionally difficult when respiratory failure persists after recovery of ventricular function. VA ECMO itself can cause pulmonary injury because of left ventricular dysfunction and disturbed aortic valve opening due to retrograde blood flow of ECMO. This may induce pulmonary edema and aortic root thrombosis [4]. Decreased pulmonary oxygenation impairs coronary and/or brain oxygen delivery when native cardiac output increases, negatively affecting an improvement in cardiac function. In such cases, transition from VA to VV ECMO via VAV ECMO may be considered to maintain oxygenation of the myocardium. Hou et al. investigated blood oxygenation in the different ECMO configurations using a sheep model of acute respiratory distress syndrome [5]. Blood oxygen saturation of the superior vena cava (41 %) was lower than that of the IVC (83 %) when using VA ECMO from the IVC to the FA. Oxygen saturation in the IVC was increased to 76 % in the VAV ECMO configuration from the IVC to the FA with an additional return cannula to the internal jugular vein. Additionally, oxygen saturation in the superior vena cava was 87 %. These findings suggest better coronary oxygenation in VAV ECMO than VA ECMO, although differential hypoxia during VA ECMO is dependent on cardiac output and pulmonary function.

Weaning from VA ECMO in pediatrics is challenging because of the following reasons. (1) Estimation for recovery in cardiac function while on VA ECMO is relatively difficult because of a lack of sufficient monitoring, including a pulmonary arterial catheter. (2) Hemodynamic support using intraaortic balloon pumping is usually inefficient because of high vascular compliance [6]. (3) Re-introduction of VA ECMO is difficult because of limited blood access. In addition, survival after second-run ECMO in children is worse compared with single-run ECMO [7]. In such cases, bridging on VAV ECMO may provide gradual and safer weaning by ensuring sufficient time to evaluate hemodynamic parameters and cardiac function [3]. Although it is not really evident, whether the transition from VA to VV ECMO via VAV ECMO was really necessary in our patient, this transition at least provided enough time for safely and definitely evaluating cardiac function in our patient. Arterial and venous return flows can be strictly titrated with the use of vascular clamps, while evaluating native cardiac function [8]. Biscotti et al. recommended setting initial blood flow to the arterial return cannula as two-thirds of the total return flow. Return flow can then be decreased depending on the native cardiac function until blood flow of 1 L/min to avoid thrombosis [8].

## Conclusions

In conclusion, we described a pediatric patient with cardiorespiratory failure who was managed with femoral VAV ECMO. VAV ECMO might be an option for safely transitioning from VA to VV ECMO by maintaining adequate cardiac oxygenation and allowing for gradual transition. Further investigations using larger cohorts are necessary to validate the efficacy of VAV ECMO as a bridging therapy in the transition from VA to VV ECMO.

## Abbreviations

ECMO, extracorporeal membrane oxygenation; FA, femoral artery; F_I_O_2_, fraction of inspired oxygen; IVC, inferior vena cava; NT-proBNP, N-terminal pro-B-type natriuretic peptide; PaCO_2_, partial pressure of carbon dioxide; PaO_2_, partial pressure of oxygen; RIJV, right internal jugular vein; VA, venoarterial; VAV, venoarterial-venous; VV, venovenous
